# Research on Deep Learning-Based Human–Robot Static/Dynamic Gesture-Driven Control Framework

**DOI:** 10.3390/s25237203

**Published:** 2025-11-25

**Authors:** Gong Zhang, Jiahong Su, Shuzhong Zhang, Jianzheng Qi, Zhicheng Hou, Qunxu Lin

**Affiliations:** 1 School of Automation, Guangdong Polytechnic Normal University, Guangzhou 510665, China; gongzhang@gpnu.edu.cn (G.Z.); zhicheng.hou@gpnu.edu.cn (Z.H.); 2 Institute for Super Robotics (Huangpu), South China University of Technology, Guangzhou 510700, China; 2230115002@smail.fjut.edu.cn; 3 School of Mechanical and Automotive Engineering, Fujian University of Technology, Fuzhou 350108, China; 2240104008@smail.fjut.edu.cn; 4 School of Rail Transportation, Wuyi University, Jiangmen 529020, China; 13427477615@163.com

**Keywords:** human-robot collaboration, dynamic and static gesture, gesture-driven control framework, deep learning, three-dimensional Convolutional Neural Networks

## Abstract

For human–robot gesture-driven control, this paper proposes a deep learning-based approach that employs both static and dynamic gestures to drive and control robots for object-grasping and delivery tasks. The method utilizes two-dimensional Convolutional Neural Networks (2D-CNNs) for static gesture recognition and a hybrid architecture combining three-dimensional Convolutional Neural Networks (3D-CNNs) and Long Short-Term Memory networks (3D-CNN+LSTM) for dynamic gesture recognition. Results on a custom gesture dataset demonstrate validation accuracies of 95.38% for static gestures and 93.18% for dynamic gestures, respectively. Then, in order to control and drive the robot to perform corresponding tasks, hand pose estimation was performed. The MediaPipe machine learning framework was first employed to extract hand feature points. These 2D feature points were then converted into 3D coordinates using a depth camera-based pose estimation method, followed by coordinate system transformation to obtain hand poses relative to the robot’s base coordinate system. Finally, an experimental platform for human–robot gesture-driven interaction was established, deploying both gesture recognition models. Four participants were invited to perform 100 trials each of gesture-driven object-grasping and delivery tasks under three lighting conditions: natural light, low light, and strong light. Experimental results show that the average success rates for completing tasks via static and dynamic gestures are no less than 96.88% and 94.63%, respectively, with task completion times consistently within 20 s. These findings demonstrate that the proposed approach enables robust vision-based robotic control through natural hand gestures, showing great prospects for human–robot collaboration applications.

## 1. Introduction

In daily practice, numerous natural behavioral communications and diverse object handovers occur between humans. With the widespread adoption of collaborative robots (Cobots), these simple yet time-consuming tasks can be seamlessly delegated to Cobots as human colleagues. Cobots can work alongside humans in a natural and efficient manner, much like human-to-human collaboration. This is known as human–robot collaboration (HRC) [1].

Due to environmental complexity, humans need to engage in diverse, efficient, safe, and natural communication with their robot teammates. Unlike verbal communication, non-verbal interaction typically relies on body language and biological signals to convey information, such as gestures [2], electroencephalography (EEG) [3], electromyography (EMG) [4], wearable sensors [5], etc. Among these, gestures are the most widely used, and gesture-based communication is crucial for achieving HRC in spacious, dynamic, and outdoor settings. Gestures also represent humanity’s most natural expressions, involving movements of the fingers, hands, head, face, or body. Leveraging machine-vision sensing technology, robots can recognize and interpret human actions in real-time, thereby enabling them to perform meaningful operations.

Human gestures can be categorized into static gestures and dynamic gestures. Static gestures [6] refer to body postures or arm positions that do not change, where the hand remains in a specific configuration even over time. Dynamic gestures [7] involve the movement of arms and hands, with their configurations changing over time. McNeill [8] further classifies gestures into four functional categories:

- Iconic gestures: Represent concrete entities or actions through visual imagery (e.g., mimicking a “drinking” motion).
- Metaphorical gestures: Convey abstract concepts indirectly by mapping physical actions to intangible ideas (e.g., spreading hands wide to signify “freedom”).
- Deictic gestures: Point to objects, directions, or spatial relationships (e.g., extending an index finger to indicate “this one”).
- Beat gestures: Synchronize with speech rhythm to emphasize timing (e.g., finger tapping to mark syllables).

This taxonomy highlights how gestures integrate spatial, temporal, and semantic dimensions to facilitate human behavioral communication.

To accurately identify and track human gestures, numerous gesture recognition methods have been proposed in recent years. Kang et al. [9] introduced a gesture recognition system that fuses Binary Neural Networks (BNNs) and lightweight Convolutional Neural Networks (CNNs), designed for classifying nine dynamic gestures rather than relatively easier-to-recognize static gestures. Agab et al. [10] proposed a vision-based system for recognizing both static and dynamic gestures. This approach employs the Dual-Tree Complex Wavelet Transform to generate approximate images with reduced noise and redundancy. The Histogram of Oriented Gradients (HOG) is then applied to these processed images to extract relevant information and produce compact feature vectors. The classification performance of three artificial neural networks was compared to four datasets consisting of alphabetic signs and dynamic gestures.

Chen et al. [11] developed a multimodal human–robot collaboration system utilizing speech and gestures for communication between humans and robots. Their approach employs Motion History Images (MHI) and deep learning (DL) methods, using Convolutional Neural Networks (CNNs) to recognize 16 dynamic gestures in real-time. Feng et al. [12] introduced YOLOv8-G2F, an enhanced network based on YOLOv8. By incorporating improved lightweight modules to replace the standard convolutional modules in both the backbone and neck, as well as the C2f module, the proposed model achieved a recognition accuracy of 99.2% on the nus-ii gesture dataset. Zhang et al. [13] developed an end-to-end, embedded gesture recognition system using low-resolution (32 × 24 pixels) infrared thermal imagery. A novel method integrating a lightweight convolutional neural network (CNN) with a Spatial Transformer Network (STN) was proposed, which achieved a recognition accuracy of 98.5% and an inference time of only 59 ms per frame on embedded devices.

Ye et al. [14] proposed a distance compensation method that integrates a 4 × 4 capacitive sensor array with an overhead ranging sensor. This approach provided a promising solution for non-contact human–computer interaction. Experimental results showed that the system achieved a gesture recognition accuracy of 93.41%, which was 5.62% higher than that achieved using capacitive sensing alone. Zhang et al. [15] presented a novel gesture recognition system utilizing LoRa technology, which incorporated advanced signal preprocessing, adaptive segmentation algorithms, and an enhanced SS-ResNet50 deep learning model. Experimental results demonstrated that the system achieves strong robustness, with an average recognition accuracy exceeding 95% for six types of gestures.

A comprehensive analysis of the aforementioned studies reveals that current gesture recognition research is primarily focused on static gestures, with dynamic gestures receiving significantly less attention. Furthermore, while most studies concentrate on comparing various solutions to improve recognition accuracy, limited attention has been given to the practical application scenarios of the recognized gestures. For instance, Adnnane et al. [16] proposed a gesture system where a glove controller with Bluetooth modules sends signals to robotic arms. However, this approach is limited to mimicking hand movements and does not enable the performance of task-oriented operations.

In particular, in human–robot collaboration, it remains a significant challenge for robots to provide accurate and real-time responses to human behaviors in terms of their temporal and spatial characteristics. To address this, this paper proposes a deep learning-based method for a human–robot gesture-driven control framework of robots, aiming to achieve grasping and the delivery of objects by leveraging static and dynamic gestures. Finally, by establishing a human–robot gesture-driven experimental platform, the two gesture recognition models are deployed onto the platform, respectively, and vision-based robot control via natural human gestures is achieved.

Our proposed deep learning-based method for static and dynamic gesture recognition enables human–robot interaction with shorter task completion times and higher success rates across varying lighting conditions and diverse participants, demonstrating robust environmental adaptability.

The rest of this study is structured as follows: Section 2 outlines the overall technical workflow, using static gestures as an example. It introduces methods for static and dynamic gesture recognition, and describes hand pose estimation and coordinate transformation. Section 3 presents experimental protocols driven by static and dynamic gestures within the proposed control framework. Section 4 provides the results and discussion. Finally, Section 5 concludes the paper and offers perspectives for future work.

## 2. Methods

### 2.1. Overall Technical Workflow

For human–robot gesture interaction, natural gestures are used to control and drive the robot to grasp and deliver objects. A two-dimensional Convolutional Neural Network (2D-CNN) [17], as well as a combined model integrating a three-dimensional Convolutional Neural Network (3D-CNN) [18] and a Long Short-Term Memory network (LSTM) [19], are trained on a custom gesture dataset to recognize unknown static gestures and dynamic gestures, respectively. The method for object 6D poses estimation incorporating hand posture [20] is adopted.

**Figure 1:**
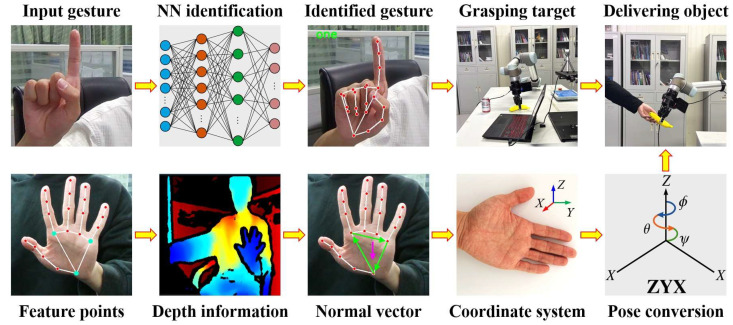
Human–robot gesture-driven overall technical workflow.

The MediaPipe [21] machine learning framework is used to extract hand feature points, which are then converted into three-dimensional coordinates to obtain the hand pose. Subsequently, by calibrating the hand–eye system, the recognized hand pose is transformed into the world coordinate system. Finally, based on the recognized gesture type, the robot determines the category of objects to grasp and deliver, thereby verifying the feasibility and effectiveness of the proposed method. Taking static gestures as an example, the overall technical workflow is illustrated in Figure 1. We collected data from four participants with varying body types and clothing, each performing 100 trials per gesture type. The results from all trials were then averaged to ensure data sufficiency and statistical reliability.

### 2.2. Static Gesture Recognition

#### 2.2.1. Static Gesture Dataset Collection

Static gestures primarily rely on limb shape and bending angles. Two common static gestures, namely “Closed fist” and “Index finger”, were selected, corresponding to controlling and driving the robot to grasp a “Bowl” and a “Banana”, respectively, as shown in Table 1.

To enhance the diversity of the static gesture data, participants of different genders, ages, and hand characteristics were invited to perform static gestures for data collection. Simultaneously, to improve the generalization capability of the gesture dataset, data collection was conducted under three distinct lighting conditions: natural light (approximately between 1000 and 100,000 lux), low light (typically below 500 lux), and high light (generally refers to levels above 10,000 lux). Five hundred images were captured for each lighting condition. During gesture data collection, participants performed the gestures in a standardized environment, and the background remained consistent to minimize the influence of interfering factors. After image collection was completed, the images underwent normalization processing, with their sizes uniformly set to 80 cm × 80 cm.

#### 2.2.2. Static Gesture Recognition Using 2D-CNN

The gesture recognition process can be divided into five steps: image acquisition, image enhancement, hand detection, feature extraction, and gesture classification. For static gestures, a single image can be used. This paper employs a two-dimensional Convolutional Neural Network (2D-CNN) [17] to train the static gesture dataset. Compared to other classification networks such as VGGNet [22], ResNet [23], and DenseNet [24], it consumes fewer computational resources during training.

**Figure 2:**
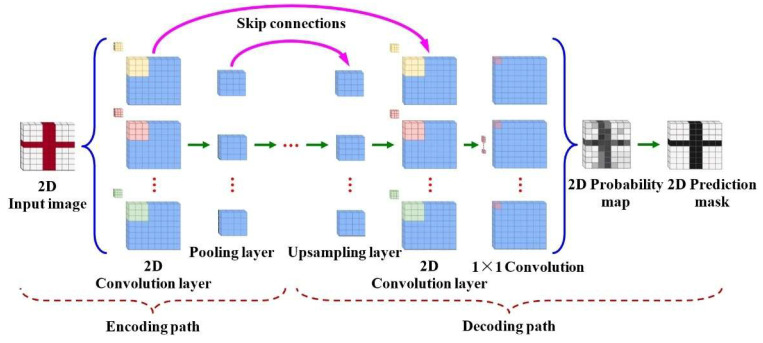
2D-CNN network architecture.

The 2D-CNN network learns spatial features from high-dimensional data space and adaptively analyzes image information without using any handcrafted features. It was successfully applied to gesture recognition problems. Its network structure is shown in Figure 2.

As can be seen from the figure, a 2D-CNN typically consists of three module layers: a convolution layer, a pooling layer, and a fully connected layer. In gesture image segmentation, it is necessary to classify each pixel simultaneously. An encoder–decoder-based Fully Convolutional Network (FCN) architecture is the best-performing model for solving segmentation problems. Most 2D data in networks use the relatively simple Euclidean representation, characterized by pixel intensity values describing each data point.

#### 2.2.3. Analysis of Static Gesture Recognition

To improve the accuracy of static gesture dataset training, this study employs a 10-fold cross-validation approach [25], dividing the original dataset into training and validation sets at a 4:1 ratio. This approach enables more comprehensive data utilization while reducing bias introduced by data partitioning, thereby yielding more reliable model evaluation results. Additionally, to enhance the model’s generalization capability, contrastive learning-based data augmentation techniques [26] are applied, including multi-cropping, rotation, and categorization, thereby increasing data diversity and improving model robustness. The network hyperparameters are configured as follows: training epochs = 60, batch size = 32, initial learning rate = 0.01, and dropout rate = 0.7, with the Adam optimizer selected.

Herein, the training accuracy of the network model reached 99.33%, demonstrating that the 2D-CNN model exhibits strong learning capabilities on the training data. The validation accuracy achieved 95.38%, indicating a certain degree of generalization ability in the network. Additionally, the training loss approached convergence by the 30th epoch, suggesting that the network model had essentially stabilized at this stage.

Following the completion of network model training, four distinct test sets were employed to evaluate model accuracy. The average recognition accuracy rates for different static gestures across these test sets were 99.05%, 98.89%, 98.61%, and 99.17%, respectively. These results demonstrate that the 2D-CNN network achieves remarkably high accuracy in static gesture recognition.

### 2.3. Dynamic Gesture Recognition

#### 2.3.1. Dynamic Gesture Dataset Collection

Unlike static gestures primarily reliant on limb shape and bending angles, dynamic gestures depend on limb trajectories, orientation, and motion velocity preserved in time series—along with shape and limb angles—thus containing richer information than their static counterparts. Using the Red–Green–Blue (RGB) mode of a depth camera, two representative dynamic gestures were captured: “Waving side-to-side” and “Backward beckoning”, corresponding to controlling and driving robotic grasping of a “Beverage can” and “Drinking cup”, respectively, as detailed in Table 2.

Same as the static gesture dataset, the collection process of dynamic gestures also covered three different lighting environments: natural light (approximately between 1000 and 100,000 lux), low light (typically below 500 lux), and high light (generally refers to levels above 10,000 lux). Participants varying in gender, age, and hand characteristics were recruited for recording sessions to enhance data diversity and generalization capability. For dynamic gestures, input images were collected as sequential frames. Since dynamic gesture recognition was sequence-dependent, each participant exhibited variations in execution speed and starting position when performing gestures.

Approximately 30 min of video streams were recorded per dynamic gesture type. Through preprocessing with video editing tools, 200 complete gesture motion clips were extracted, with each gesture category containing around 100 video clips. Each video clip lasts approximately 1 s, comprising roughly 30 effective frames that comprehensively preserve the temporal characteristics of dynamic gestures. To ensure data integrity, all collected images were detected using the handdetect function from the PyCharm 4.5.4 library. This verification step prevented cases where gestures are completely absent or only minimal portions of gestures exist in some images.

To extract representative gesture images from each gesture category for learning, a buffer queue length of 20 was established to temporarily store incoming gesture images. Upon collecting 20 images, these were preprocessed and normalized to a uniform size of 64 cm × 48 cm for subsequent recognition and classification tasks.

#### 2.3.2. Dynamic Gesture Recognition via Hybrid 3D-CNN + LSTM Architecture

For dynamic gesture recognition, relying solely on temporal or spatial features is insufficient. Instead, fusion of spatial and temporal features is essential. The 3D Convolutional Neural Network (3D-CNN) [18], capable of extracting spatiotemporal features, offers superior performance for such tasks by leveraging the strengths of 2D-CNNs [17] for image classification while incorporating temporal features. This approach preserves spatial information across video frames to capture temporal dynamics. The 3D-CNN architecture processes three-dimensional volumetric data as input and generates similarly sized three-dimensional binary masks as output.

**Figure 3:**
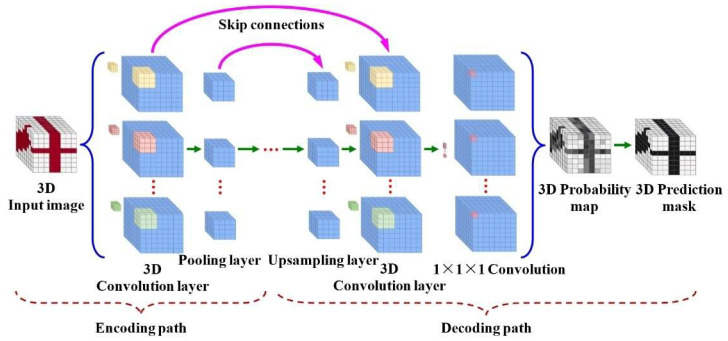
3D-CNN network architecture.

The network architecture of 3D-CNN resembles a standard 2D-CNN (as shown in Figure 2) but employs 3D extensions in its convolutional and pooling layers to process volumetric data. Specifically, convolutional layers perform filtering using three-dimensional kernels, while pooling layers downsample data within 3D spatial dimensions to compress feature space size. This hierarchical processing progressively analyzes volumetric data into cuboidal patches, enabling comprehensive learning of 3D spatial features as illustrated in Figure 3. By retaining spatial correlations while capturing motion trajectories, 3D-CNNs effectively address the challenges of dynamic gesture recognition, such as occlusions and variable speeds.

As illustrated in the diagram, the 3D-CNN architecture typically comprises an encoding path and a decoding path. Within the encoding path, 3D convolutional layers and pooling layers collaboratively construct a 4D feature space. The number of filters employed in each 3D convolutional layer determines the dimensionality of the fourth channel dimension, leading intermediate 3D convolutional layers to perform 4D convolution operations.

Since pooling progressively reduces feature space dimensionality along the encoding path, the decoding path utilizes a corresponding number of upsampling layers to incrementally restore spatial dimensions comparable to the input data. The final layer employs (1 × 1 × 1) convolution to project stacked features into a space matching the input volume dimensions. Subsequently, a SoftMax function generates 3D probability maps, which undergo thresholding to yield the ultimate 3D prediction mask.

Although 3D-CNNs can extract temporal features while preserving the spatial characteristics of gesture images, they are limited to capturing only short-term temporal dependencies. Given that most dynamic gestures span 32–50 frames, relying solely on 3D-CNNs proves insufficient for learning long-range spatiotemporal information from video datasets. This limitation necessitates integrating an additional network architecture capable of modeling long-term temporal dynamics.

To address this, we introduce Long Short-Term Memory (LSTM) networks [19] to resolve extended and complex sequence challenges in dynamic gesture recognition. LSTM networks can learn long-term dependencies through their sophisticated architecture, which incorporates input, output, and forget gates to regulate long-term sequence pattern learning. These gates are modulated by sigmoid functions that learn to control their open/closed states during training. By combining 3D-CNNs for spatial–temporal feature extraction and LSTMs for sequence modeling, the framework could achieve robust performance in dynamic gesture recognition tasks.

**Figure 4:**
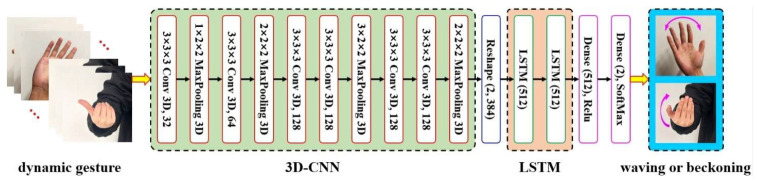
3D-CNN+LSTM hybrid network architecture.

To this end, this paper proposes a hybrid network architecture integrating 3D-CNN and LSTM networks to extract local and global features from video sequences, respectively. The hybrid network architecture comprises two fundamental components: a 3D convolutional module extracting local spatiotemporal features from gesture sequences through 3D convolution and pooling operations, and an LSTM module capturing global spatiotemporal features, as illustrated in Figure 4. The fusion of these modules enhances the model’s ability to recognize complex gestures involving both sustained gestures (e.g., closed fist) and rapid motions (e.g., waving side-to-side).

As shown in the diagram, we introduce a Reshape layer between the 3D-CNN and LSTM networks. The core function of the Reshape layer is to reorganize the 4D features output by the 3D-CNN into a time-sequential 2D format compatible with LSTM input requirements, while normalizing the second dimension (384-dimensional feature vectors), which is equivalent to performing unitization on the feature vector at each time step.

The input tensor for the 3D-CNN+LSTM network is structured as [20, 48, 64, 3]. The first dimension represents the 20-frame image sequence, indicating the number of samples processed per training iteration. The values 48 and 64 correspond to the height and width of each frame, respectively, while 3 indicates the three channels of the RGB color space.

The core processing pipeline operates as follows: First, the convolutional layers (Conv 3D) within the 3D-CNN process the dynamic gesture image sequence, which inherently includes the temporal dimension, to extract both spatial and temporal features of the gestures. Downsampling is then performed using MaxPooling 3D layers, which reduces data dimensionality while preserving critical features, thereby lowering computational complexity. Subsequently, a Reshape operation adjusts the data dimensions, preparing the processed feature data for input into the LSTM network. The LSTM network captures the temporal dependencies within the dynamic gesture sequence and further processes the features. Following this, fully connected layers (Dense) integrate and transform the features output by the LSTM. Finally, a SoftMax layer produces the classification results, enabling the recognition of dynamic gestures.

#### 2.3.3. Analysis of Dynamic Gesture Recognition

To enhance the generalization capability of the 3D-CNN+LSTM network architecture, contrastive learning-based data augmentation techniques [26] are similarly employed as with the static gesture-processing approach described earlier. This includes techniques such as random cropping, random center cropping, corner cropping, and scale jittering. The hyperparameters for network training are set as follows: training epochs = 30, batch size = 20, and learning rate = 0.015, with Adam optimizer likewise selected.

On a custom dynamic gesture dataset, the 3D-CNN+LSTM network architecture initially exhibited a training loss of approximately 1.4. However, by the 10th epoch, the loss rapidly decreased to below 0.2, indicating swift data features learning during the early training phase. By the 30th epoch, the training loss reached convergence. The validation loss eventually stabilized around 0.15, remaining close to the training loss without significant divergence. This demonstrates no severe overfitting and reflects the model’s strong generalization capability.

Additionally, both the training accuracy and validation accuracy of the proposed network model showed an upward trend. The final training accuracy reached 95.66%, while the validation accuracy achieved 93.18%. Although the training accuracy slightly surpassed the validation accuracy, the marginal gap (2.48 percentage points) indicates robust generalization performance when processing unseen data.

To further validate the accuracy of the proposed 3D-CNN+LSTM network architecture for dynamic gesture recognition, we conducted comparative experiments on a custom dynamic gesture dataset against three other neural network models, as detailed in Table 3. The comparison results in Table 3 demonstrate that for recognizing identical dynamic gestures, our hybrid 3D-CNN+LSTM network architecture outperforms typical network models—C3D [27], Res3D [28], and 3D CNN-RNN [29]—in both computational efficiency and recognition accuracy.

### 2.4. Hand Pose Estimation and Coordinate Transformation

#### 2.4.1. Hand Pose Estimation

Object pose estimation is a critical task in computer vision and robotics, aiming to determine the position of the hand in three-dimensional space when performing corresponding gestures. Feature point-based pose estimation methods [30], which extract local feature points of the target, form the foundation of pose measurement. However, the coordinate data of these feature points inevitably contain measurement errors. Directly estimating the pose without distinguishing the measurement errors of individual points will cause the estimated value to deviate from the true value. The MediaPipe [21] machine learning framework enables the extraction of key human body feature points in complex environments. It can better handle the complex interference effects (such as rotation, scale, and lighting variations) on static and dynamic gestures, achieving 2D–3D matching and solving for hand pose.

**Figure 5:**
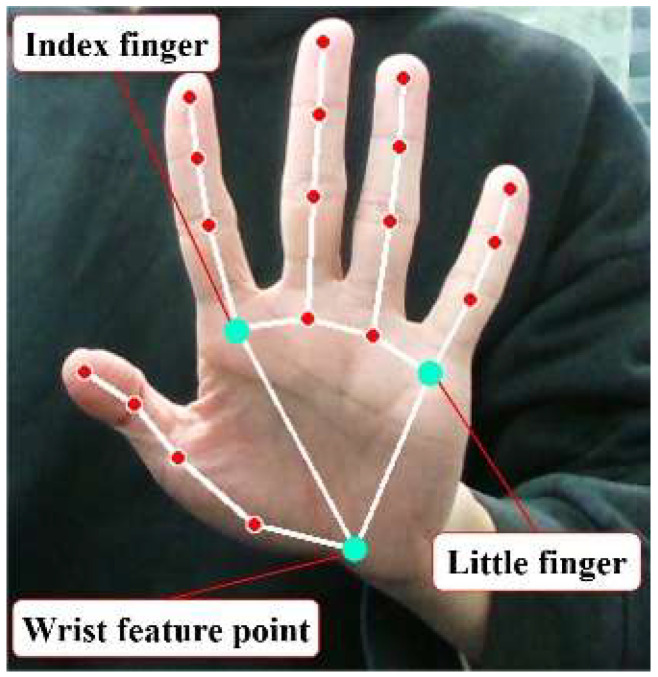
Three hand key points.

First, the hand detection object is initialized, configured to process information for only a single hand at a time. Furthermore, it requires the confidence score of the detected hand landmarks to exceed 70%, ensuring the reliability of the detection results. Since MediaPipe [21] requires input images to be in RGB format, each gesture video frame must first be converted to RGB format. It is then processed by the hand process (image) function to obtain the hand key-point information. Hand pose estimation primarily involves three key points: the base of the index finger, the base of the little finger, and the wrist area, as shown in Figure 5.

**Figure 6:**
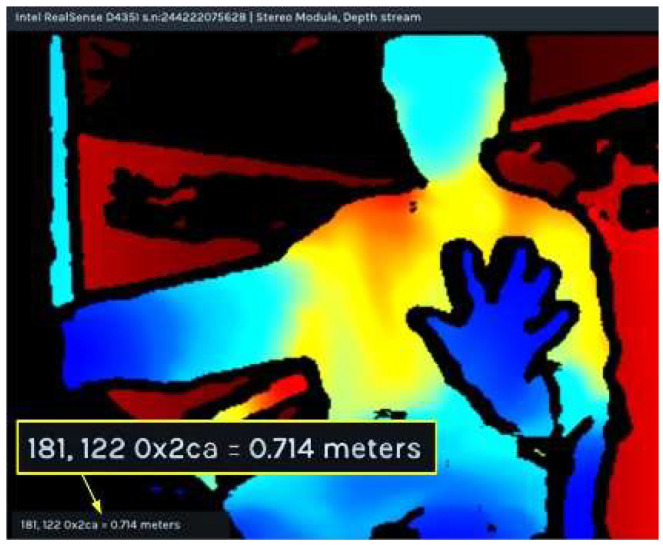
Hand depth image.

Next, we will convert the 2D feature points of the hand into 3D coordinates. First, we acquire the depth image information of the hand—specifically, the distance from the hand to the depth camera—as illustrated in Figure 6. The point (188, 122) shown in the image represents pixel coordinates, 0 × 2 ca is the raw depth data value, and 0.714 m corresponds to the actual geometric distance between the camera and the hand position at that pixel.

After extracting 2D key points from hand RGB images using MediaPipe [21], triangulation-based methods [31] are applied. Combined with camera intrinsics and depth images, this enables the conversion of 2D key points into 3D coordinates.

**Figure 7:**
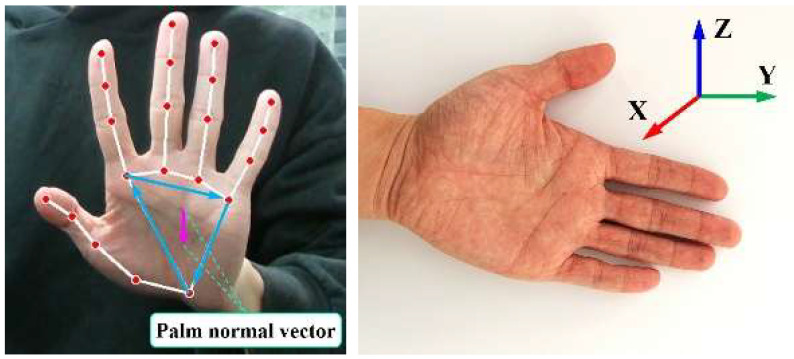
The three vectors of the hand and the hand orientation coordinate system for (**left**) the three-key-point vector, and (**right**) the hand orientation coordinate system.

Next, the rotation matrix characterizing finger orientation is calculated using the vector cross-product method [32]. The normal vector of the palm can be determined by computing the cross-product of vectors formed by three key points on the palm (including the wrist, base of the index finger, and base of the little finger). The vectors constructed from these three key points are depicted in Figure 7 (left).

Subsequently, the computed normal vector is normalized. Then, based on the relationship between the normalized vector and other basis vectors, three axial vectors (including *x*-axis, *y*-axis, and *z*-axis) of the rotation matrix are derived, thereby obtaining the hand orientation coordinate system. This yields the rotation matrix representing hand orientation, as shown in Figure 7 (right).

#### 2.4.2. Hand–Eye Calibration and Pose Transformation

Calibration is indispensable in vision-based human–robot gesture-driven control, and its precision determines the accuracy of robotic grasping and delivery of objects. Starting with an analysis of camera imaging principles, this paper conducts camera intrinsic parameter calibration and robot hand–eye calibration for the human–robot gesture-driven system. It transforms object poses recognized in neural networks into positions and orientations in the world coordinate system for conducting human–robot gesture-driven experiments. To solve the transformation relationship between the robot base coordinate system and the camera coordinate system, the Eye-to-Hand configuration [33] is adopted. In this setup, the camera is mounted on a fixed base separate from the robot and does not move with the robotic arm.

Setting: The camera coordinate system is denoted as $O_{c}$, the calibration board coordinate system as $O_{w}$, the robot end-effector coordinate system as $O_{e}$, and the robot base coordinate system as $O_{b}$. The pose transformation from the calibration board coordinate system to the camera coordinate system is $T_{w}^{c}$, from the camera coordinate system to the robot base coordinate system is $T_{c}^{b}$, and from the robot base coordinate system to the robot end-effector coordinate system is $T_{b}^{e}$.

During calibration, the calibration board is rigidly mounted to the robot end-effector to ensure its relative pose remains constant. When the robot moves to a specific pose, the coordinates of a given point on the calibration board in the $O_{w}$ coordinate system are denoted as $P_{1}$, while its coordinates in the robot end-effector coordinate system $O_{e}$ are $P_{3}$. Thus, the initial position can be expressed as follows:

(1) $$T_{b}^{e}T_{c}^{b}T_{b}^{e}P_{1}=P_{3}$$

After the robot transforms its pose, the above transformation can be expressed as follows:

(2) $$T_{b}^{e^{\prime }}T_{c}^{b}T_{w}^{c^{\prime }}P_{1}=P_{3}$$

We obtain by simultaneously transforming Equations (1) and (2):

(3) $$T_{b}^{e}T_{c}^{b}T_{w}^{c}P_{1}=T_{b}^{e’}T_{c}^{b’}T_{w}^{c’}P_{1}$$

By further transforming, we can yield the following:

(4) $$T_{b}^{e^{\prime }-1}T_{b}^{e}T_{c}^{b}=T_{c}^{b}T_{w}^{c^{\prime }}T_{w}^{c-1}$$

Through iterative refinement of the robot’s end-effector pose, the aforementioned equations can be solved to obtain the value of the target matrix $T_{c}^{b}$. This paper utilizes the ros_aruco package in ROS (Robot Operating System) to acquire the transformation $T_{w}^{c}$ between the calibration board coordinate system and the camera coordinate system. The pose transformation $T_{b}^{e}$ from the robot base coordinate system to the end-effector coordinate system is obtained through URScript of Universal Robots. Substituting these into the Tsai–Lenz algorithm [34], the pose transformation $T_{c}^{b}$ from the camera coordinate frame to the robot base coordinate frame is solved, with results presented in Equation (5):

(5) $$T_{c}^{b}=\left[\begin{array}{cccc} -0.0348 & 0.5627 & -0.8200 & 0.4702\\ 0.9990 & 0.0122 & -0.0300 & -0.4920\\ -0.0080 & -0.8250 & -0.5628 & 0.9831\\ 0 & 0 & 0 & 1 \end{array}\right]$$

After obtaining this transformation matrix, it is used to transform the estimated hand pose from the camera coordinate system to the robot coordinate system.

## 3. Experimental Protocols

**Figure 8:**
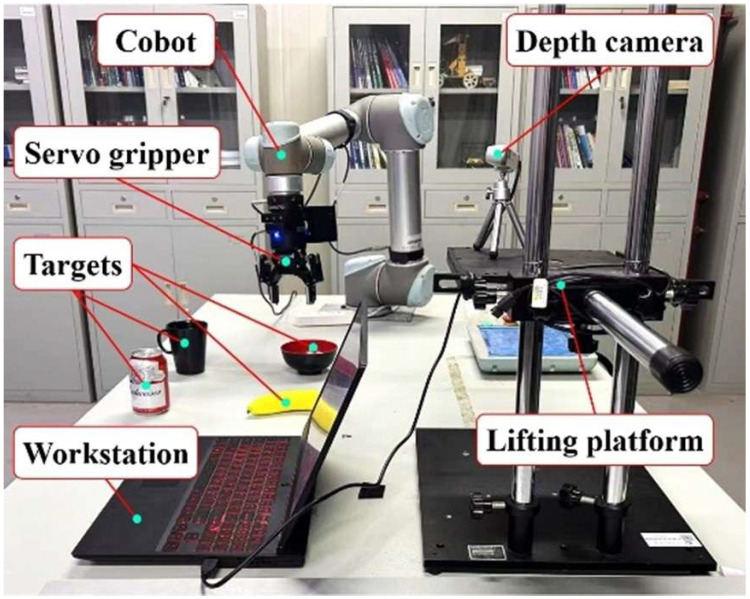
Human–robot static/dynamic gesture-driven experiment platform.

The experimental platform for human–robot static and dynamic gesture-driven experiments primarily consists of the following: a Cobot (Universal Robots, Odense, Denmark, UR5) featuring a 5 kg payload and ±0.03 mm repeatability; a servo gripper (ROBOTIQ, Lévis, QC, Canada, 2F-85) with an 85 mm stroke and 20–235 N gripping force range; a stereo depth camera (Intel, Santa Clara, CA, USA, RealSense D435i) offering <2% depth error at 2 m and 1920 × 1080 @30 fps RGB video; a high-performance mobile workstation (Lenovo, Beijing, China, ThinkBook16P) equipped with an Ultra 9 275HX CPU and NVIDIA GeForce RTX 4060 GPU; and a lifting platform, as illustrated in Figure 8.

The depth camera D435i is primarily used to collect datasets of static and dynamic gestures. It is equipped with a stereo depth module, an RGB sensor, and an inertial measurement unit (IMU) for capturing six-degree-of-freedom (6D) data. It can then recognize and classify static/dynamic gestures in real-time and perform hand pose estimation by collecting RGB and depth images of the hand, which is intended for subsequent robot grasping and delivery operations. The lifting platform enables height adjustment of the depth camera to ensure precise gesture recognition and hand pose estimation for participants of varying statures.

We employed a diverse dataset to improve gesture recognition generalization. Data from four participants with varying body types and clothing were collected across three lighting conditions (natural, low, and strong light), with each participant performing 100 trials per gesture type. The operational workflow for human–robot static/dynamic gesture-driven control is as follows: The high-performance mobile workstation captures target hand data from the depth camera, then recognizes and classifies these gestures. Following the algorithmic processing described previously, the system outputs real-time poses, converts these poses into the robot end-effector coordinate system, and ultimately controls the Cobot to grasp objects and deliver them to designated locations.

## 4. Results and Discussion

**Figure 9:**
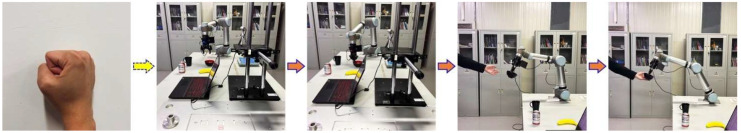
The static gesture “closed fist” drives the robot to grasp and deliver a “bowl”.

**Figure 10:**
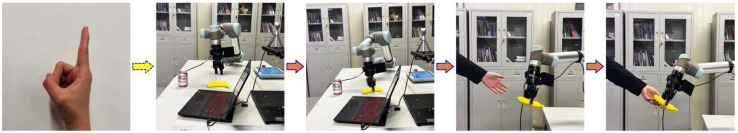
The static gesture “index finger” drives the robot to grasp and deliver a “banana”.

For two common static gestures, when participants face the depth camera and perform the “closed fist” and “index finger” gestures, respectively, the human–robot gesture-driven system will recognize these corresponding gestures, conduct hand pose estimation, and subsequently transmit the transformed pose to the robot. The robot then grasps a “bowl” and a “banana”, respectively, delivering them to the participant’s hand. The typical procedure comprises five stages: showing gesture, recognizing, awaiting grasp, grasping, and delivery, as depicted in Figure 9 and Figure 10.

**Figure 11:**
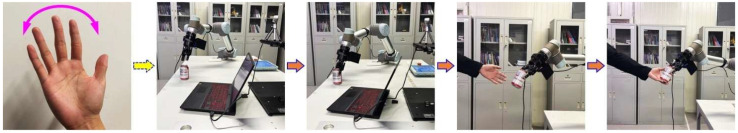
The dynamic gesture “waving side-to-side” drives the robot to grasp and deliver a “beverage can”.

**Figure 12:**
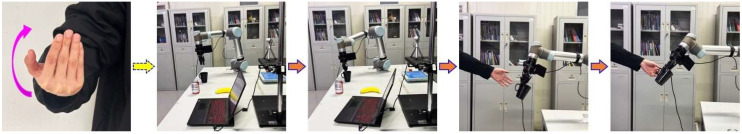
The dynamic gesture “backward beckoning” drives the robot to grasp and deliver a “drinking cup”.

Similarly, as for two common dynamic gestures, when participants face the depth camera and perform “waving side-to-side” and “backward beckoning” gestures, respectively, the human–robot gesture-driven system will recognize these corresponding gestures, conduct hand pose estimation, and subsequently transmit the transformed pose to the robot. The robot then grasps a “beverage can” and a “drinking cup”, respectively, delivering them to the participant’s hand. The standardized procedure comprises the same five stages: showing gesture, recognizing, awaiting grasp, grasping, and delivery, as illustrated in Figure 11 and Figure 12.

To comprehensively evaluate the robustness of the human–robot static/dynamic gesture-driven system under varying lighting conditions, this study employs two evaluation metrics: task completion time and task success rate. Comparative experiments were conducted in three lighting conditions: natural light (approximately between 1000 and 100,000 lux), low light (typically below 500 lux), and high light (generally refers to levels above 10,000 lux). Four participants with varying body types and clothing were selected. Each participant performed 100 gesture-driven trials for both gesture types, with results averaged to ensure data sufficiency and statistical reliability. For participant safety, the robot end-effector speed was limited to 0.5 m/s during experiments. The performance of two static gestures under three lighting conditions is presented in Table 4.

As evidenced by Table 4, static gestures consistently achieved high average success rates in controlling and driving the robot to complete grasping and delivery tasks across all three lighting conditions. The lowest success rate was 96.88%, while the highest reached 98.13%, with overall fluctuations within 2%. This demonstrates significant robustness of the human–robot static/dynamic gesture-driven system against lighting variations. Furthermore, the average task completion time ranged between 17.60 s and 18.43 s under different lighting conditions, with even less variation observed between different gestures under identical lighting. This indicates stable response times of the human–robot static gesture-driven system across varied gestures.

Similarly, the performance of two dynamic gestures under three lighting conditions can be obtained, as represented in Table 5.

As shown in Table 5, the overall success rate of dynamic gestures across three lighting conditions is slightly lower than that of the static gesture system. The primary reason lies in the requirement for continuous recognition across multiple frames in the temporal sequence for dynamic gestures, where variations in gesture motion amplitude may also affect complete recognition. Nevertheless, the average success rate remains above 94.63%, with a maximum variance not exceeding 1%, indicating a limited impact of lighting variations on dynamic gesture recognition. Furthermore, the average task completion time ranges from 18.38 s to 19.42 s under different lighting conditions, with temporal differences within 1.1 s.

The robustness of gesture recognition systems is adversely affected by variations in illumination conditions. Phenomena including excessive brightness, inadequate lighting, shadows, and uneven illumination can compromise the apparent visual attributes of the hand, such as its color, texture, and contour geometry, thereby inducing performance deterioration or outright failure of the recognition model. Furthermore, a pronounced intra-class variability is inherent to gestures, wherein identical gestures may display markedly different appearances owing to inter-user disparities in hand dimensions, morphology, and nuanced articulatory nuances.

However, the experimental results above confirm that our proposed deep learning-based method for static and dynamic gesture-driven human–robot interaction completes tasks more quickly and with greater success, even under challenging variations in lighting and across a diverse user group. This performance translates directly into robust environmental adaptability, indicating significant potential for deployment in human–robot collaborative applications.

## 5. Conclusions

To achieve efficient and natural human–robot interaction, this paper investigates deep learning-based static/dynamic gesture-driven robotic technology. A 2D-CNN and a 3D-CNN+LSTM are employed for static and dynamic gesture recognition, respectively. Subsequently, hand pose estimation was performed to control and drive the robot in grasping and delivering objects. This was integrated with a depth camera-based method for 2D-to-3D coordinate conversion of feature points to estimate poses. Through camera intrinsic calibration and robot hand–eye calibration, coordinate system transformation was realized, obtaining hand poses in the robot base coordinate system and converting them to the manipulator coordinate system. Finally, an experimental platform for human–robot gesture-driven control was established. Experiments with diverse participants under varying lighting conditions demonstrate that both gesture systems achieved average response times below 20 s and success rates exceeding 94%, indicating good applicability. This demonstrates significant practical value for human–robot collaboration scenarios.

In this study, model deployment and application face computational bottlenecks. Although the high-precision 3D CNN-based model delivers excellent gesture recognition performance, its high computational complexity makes it difficult to achieve real-time inference on resource-constrained embedded devices such as smartphones. Consequently, we must strike a balance between gesture recognition accuracy and gesture-processing speed. A feasible solution involves leveraging mobile GPUs or dedicated edge AI chips for hardware acceleration, along with frameworks like TensorRT and TFLite for optimized on-device deployment.

Given that in practical applications, variations in users’ hand characteristics and gesture speeds can interfere with gesture recognition systems, future research could explore the integration of multimodal learning—incorporating visual, tactile, and auditory information—or leverage depth sensors and haptic feedback. These approaches would enable robots to better understand users’ motion intentions, thereby improving the robustness of gesture-driven systems.

## Figures and Tables

**Table 1:** Static gesture categories and their corresponding objects.

Gesture Name	Gesture Example	Target Object	Object Name
Closed fist	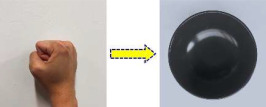		Bowl
Index finger	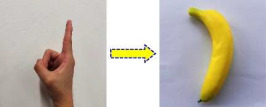		Banana

**Table 2:** Dynamic gesture categories and their corresponding objects.

Gesture Name	Gesture Example	Target Object	Object Name
Waving side-to-side	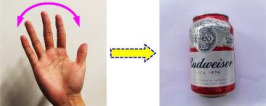		Beverage can
Backward beckoning	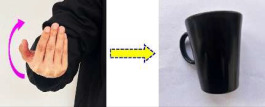		Drinking cup

**Table 3:** Performance comparison of four network models.

Model Name	Parameter Count	Recognition Accuracy
C3D [27]	94,910,722	92.73%
Res3D [28]	26,547,498	94.49%
3D-CNN+RNN [29]	3,688,706	94.85%
3D-CNN+LSTM	5,547,143	95.66%

**Table 4:** The performance of two static gestures under three lighting conditions.

Participants	Natural Light		Low Light		High Light	
	Completion Time/s	Success Rate/%	Completion Time/s	Success Rate/%	Completion Time/s	Success Rate/%
1	18.18	98.50	19.22	95.00	18.93	97.00
2	18.13	98.00	18.22	97.00	18.20	99.50
3	17.28	99.00	18.55	96.50	19.69	94.50
4	16.84	99.00	17.91	98.00	16.79	99.00
Average value	17.60	98.13	18.43	96.88	18.39	97.63

**Table 5:** The performance of two dynamic gestures under three lighting conditions.

Participants	Natural Light		Low Light		High Light	
	Completion Time/s	Success Rate/%	Completion Time/s	Success Rate/%	Completion Time/s	Success Rate/%
1	17.94	96.50	19.95	94.50	19.71	95.50
2	18.70	95.00	18.69	95.50	19.08	95.50
3	18.68	95.50	19.44	95.00	19.49	95.00
4	18.21	96.50	19.60	93.50	19.27	94.50
Average value	18.38	95.88	19.42	94.63	19.39	95.13

## Data Availability

The data presented in this study are openly available in [Research on Deep Learning-Based Human–Robot Static/Dynamic Gesture-Driven Control Framework—research data] at [https://www.weiyun.com/disk/folder (accessed on 1 November 2025)].
